# Draft genome sequence of the mammalian pathogenic *Pythium insidiosum*

**DOI:** 10.1128/mra.00445-26

**Published:** 2026-10-26

**Authors:** Maiara Goncalves Ramos, Fahong Yu, Rebecca Barber, Rebecca Nance-Richey, Sara D. Lawhon, Jing Wu, Catherine G. Davis, Sara Green, Erica M. Goss, Domenico Santoro, Aline Rodrigues Hoffmann

**Affiliations:** 1 Comparative, Diagnostic, and Population Medicine, College of Veterinary Medicine, University of Florida 70122, Gainesville, Florida, USA; 2 Interdisciplinary Center for Biotechnology Research, University of Florida 3463, Gainesville, Florida, USA; 3 Small Animal Clinical Sciences, Microbiology Laboratory, College of Veterinary Medicine, University of Florida 70122, Gainesville, Florida, USA; 4 Small Animal Clinical Sciences, College of Veterinary Medicine, University of Florida 70122, Gainesville, Florida, USA; 5 Veterinary Pathobiology, College of Veterinary Medicine and Biomedical Sciences, Texas A&M University 14736, College Station, Texas, USA; 6 Veterinary Medical Teaching Hospital, College of Veterinary Medicine and Biomedical Sciences, Texas A&M University 14736, College Station, Texas, USA; 7 Plant Pathology Department, Institute of Food and Agricultural Sciences and Emerging Pathogens Institute, University of Florida 316814, Gainesville, Florida, USA; University of California Riverside, Riverside, California, USA

**Keywords:** oomycetes, mammalian oomycetes, genomes, characterization, pythiosis

## Abstract

The reported genome represents a nearly complete reference assembly of the mammalian pathogenic oomycete *Pythium insidiosum*, generated from clinical isolates collected from animals in the southern United States. Genome assembly was performed from combined long- and short-read sequencing data obtained with the Oxford Nanopore Technologies and Illumina MiSeq platforms.

## ANNOUNCEMENT

*Pythium insidiosum* causes life-threatening disease in humans and animals (1, 2) with poor response to antifungal therapy (1, 3, 4). Genomic characterization of mammalian isolates facilitates understanding of virulence and improves diagnostic strategies. Clinical isolates from a dog in Texas (7011-1) and from a horse and a dog in Florida (111-60 and 111-70) were submitted to our diagnostic service for suspected oomycosis. The original samples were plated on Columbia blood agar (Thermo Fisher Scientific, USA) at 35°C for 48–72 h, and the leftover material was used, with the owner’s consent, to obtain pure cultures by subculturing mycelium onto fresh Columbia plates. Mycelia from each isolate were individually collected for DNA extraction using the Wizard Genomic DNA Purification Kit (Promega) according to the manufacturer’s protocol. DNA concentration was assessed by Qubit and quality by NanoDrop and TapeStation. The pathogen’s identity was confirmed through PCR amplification of the internal transcribed spacer 2 (ITS) and large subunit (28S) regions on DNA isolates. Results were Sanger-sequenced (Applied Biosystems, 3730xl Genetic Analyzer) and matched 100% (ITS) and 99.90% (28S) to *Pythium insidiosum* in GenBank (5). Short-read sequencing was initially generated for assembly accuracy using isolate 7011-1. Long reads were later obtained from Florida isolates to improve contiguity and better resolve the genomic heterogeneity typical of oomycetes. A 2,332 ng gDNA Illumina library was prepared, purified with AMPure XP beads, and sequenced on an Illumina MiSeq (v2 chemistry, Illumina Inc., San Diego, CA, USA) to generate 2 × 250 bp paired-end reads. The resulting 152,949,22 raw reads were quality-trimmed using Cutadapt (3.4) (6) and assessed with FastQC (v0.12.1) (7). Isolates 111-60 and 111-70 were sequenced independently on a GridION (R10.4.1 flow cell, Oxford Nanopore Technologies [ONT]) in 400 bp mode. Isolate 111-70 was sequenced twice to increase total sequencing yield. Nanopore libraries were prepared using the ONT PCR-free Ligation Kit (SQK-NBD114.96) and NEBNext Companion Module (E7180L). Sequencing generated 5,230,003 reads (N50 = 2,396 bp), including all 3 read sets from 111-60 and 111-70 isolates. Dorado (v0.5.3) (8) performed basecalling using the super-accurate model and 5mC/5hmC detection, and FASTQ files were generated with Samtools fastq (v1.20) (9). Adapter trimming was performed during demultiplexing, and sequencing quality statistics were generated using fastQC (v0.23.4) (10). Porechop (v0.2.4) (11) was applied to filter low-quality bases prior to assembly. The clean ONT reads were combined and assembled *de novo* using Flye v2.9.3 (12). The resulting assembly was polished with Medaka v2.0 (13) using ONT reads and subsequently refined with two rounds of Pilon (14) polishing using Illumina reads from 7011-1, to improve contiguity and base-level accuracy (15, 16). Genome annotation was completed using the following software: Funannotate (v1.8.17) (17) with Augustus (*Toxoplasma gondii*) (18), InterPro (iprscan/5.75–106.0) (19), eMapper (eggnog-mapper/2.1.12) (20), QUAST (v5.3.0) (21), and BUSCO (v5.8.3, eukaryota_odb10) (22). Genome completeness was assessed using BUSCO and estimated at 91.4% based on the recovery of conserved single-copy orthologs. Detailed statistics for the genome assembly and annotation are summarized in Table 1.

**TABLE 1:** Summary statistics for the *Pythium insidiosum* genome assembly and annotation

Characteristics	Value
Total assembly length (Mb)	43.4
No. of scaffolds	1,219
No. of contigs (≥1,000 bp)	1,067
No. of contigs (≥5,000 bp)	640
No. of contigs (≥10,000 bp)	498
Largest contig	793,719
N50 (bp)	177,225
L50	70
Total BUSCO groups	255
No. of complete BUSCOs (%)	91.4%
No. of complete single-copy BUSCOs (%)	90.2%
No. of duplicate single-copy BUSCOs (%)	1.2%
No. of incomplete BUSCOs (%)	1.6%
No. of missing BUSCOs (%)	7.1%
%GC	58
Total no. of predicted protein-coding genes	13,947
NCBI BioProject accession no.	PRJNA1334412
GenBank assembly accession no.	JBVVWS000000000

## Data Availability

This genome sequence of Pythium insidiosum has been deposited in the National Center for Biotechnology Information (NCBI) under BioProject PRJNA1334412 and the accession JBVVWS000000000. The raw sequencing reads have been deposited in the NCBI Sequence Read Archive under accession numbers SRR37700723 and SRR37700724. Sanger sequences generated in this study have been deposited in GenBank under accession numbers PZ761169–PZ761171 (ITS) and PZ761200–PZ761202 (28S).
